# Platelet-dependent thrombography gives a distinct pattern of in vitro thrombin generation after surgery with cardio-pulmonary bypass: potential implications

**DOI:** 10.1186/1477-9560-10-15

**Published:** 2012-08-21

**Authors:** Rose Said, Véronique Regnault, Marie Hacquard, Jean-Pierre Carteaux, Thomas Lecompte

**Affiliations:** 1 Laboratoire d’Hématologie et Institut Cardiovasculaire Nancy, Centre Hospitalier Universitaire, Nancy, France; 2 Inserm U961, Nancy Université, Nancy, France; 3 EFS Lorraine Champagne, Nancy, France; 4 Haematology Laboratory, CHU Nancy, Rue du Morvan, 54511, Vandoeuvre les Nancy Cedex, France

**Keywords:** Cardio pulmonary bypass, Thrombin, Platelets, Thrombography

## Abstract

**Background:**

Bleeding remains a potentially lethal complication of cardio-pulmonary bypass (CPB) surgery. The purpose of this study was to obtain a better insight into *in vitro* thrombin generation in the context of CPB.

**Methods:**

We used Calibrated Automated Thrombography to assess blood coagulation of 10 low-risk patients operated for valve replacement with CPB, under 2 experimental conditions, one implicating platelets as platelet dysfunction has been described to occur during CPB.

**Results:**

Our main finding was that CPB-induced coagulopathy was differently appreciated depending on the presence or absence of platelets: the decrease in thrombin generation was much less pronounced in their presence (mean endogenous thrombin potential change values before and after CPB were -3.9% in the presence of platelets and -39.6% in their absence).

**Conclusion:**

Our results show that experimental conditions have a profound effect in the study of *in vitro* thrombin generation in the context of CPB.

## Introduction

Bleeding remains a feared complication of cardiopulmonary bypass (CPB) surgery. Despite improvements, blood coagulation is still altered in the extracorporeal circuit [1-4]. We have reported in variant haemophiliacs that Calibrated Automated Thrombography (CAT), as an integrative *in vitro* phenotyping of coagulation, would be a better laboratory approach to assess the bleeding risk than the assays currently used in laboratory medicine [5]. Recent works have studied CAT thrombin generation in plasma of patients after CPB. Schols *et al.*[6] showed a decrease in thrombin generation associated with a decrease in fibrin formation (assessed with thromboelastography). In addition, Solomon *et al*[7] have shown that fibrin formation is more altered than thrombin generation following CPB. Furthermore, Coakley *et al.*[8] have shown a correlation between pre- and post-operative Endogenous Thrombin Potentials (ETP), and more importantly an association with bleeding complications, suggesting that this assay is informative in this context [5]. CAT can be performed in the presence of platelets [9]. To obtain a better insight into *in vitro* thrombin generation in the context of CPB, we used CAT under 2 experimental conditions, one implicating platelets. To the best of our knowledge this has never been performed with CAT in the context of CPB.

## Materials and methods

### Patient population

Ten low-risk adult patients scheduled for replacement of one heart valve (6 aortic, 4 mitral) included. There were 7 men and 3 women; 70 years (59-87) of age (continuous variables are expressed as medians and ranges). All patients signed an informed consent form approved by the local research committee.

### Collection of blood samples

Venous blood samples were collected before CPB and shortly after protamine injection into S-Monovette® tubes (Sarstedt) containing 0.109 M citrate, in the proportion of 1 volume citrate to 9 volumes of blood. Platelet-Rich Plasma (PRP) was prepared by blood centrifugation at 190 g for 10 min at 20°C. The PRP was recovered and adjusted to 150 G/L by the addition of Platelet-Poor Plasma (PPP) obtained by centrifugation of the remaining blood at 1750 g for 10 min at 20°C. Platelet- depleted Plasma (PDP) was obtained from PPP centrifuged at 13000 g for 30 min at 4°C. PDP was immediately frozen at -80°C and thawed 15 min at 37°C when needed.

### Thrombin generation

CAT was performed with PRP and PDP as well, as described in [10]. Measurements were carried out in 96-well circular bottom microtiter polypropylene plates (Greiner). Coagulation was initiated with 0.3 (PRP and PDP), 1 or 5 pM (PDP only, for practical reasons) recombinant human tissue factor (TF) reagent (Innovin®, Dade Behring), and, when PDP was studied, in the presence of 4 μM phospholipid vesicles: phosphatidylcholine (PC)/ phosphatidylserine (PS)/ phosphatidylethanolamine (PE); (PC/PS/PE, 60/20/20, mole %).

### Statistical analysis

Data are presented as median (min-max). Wilcoxon test was used to compare before and after surgery coagulation parameters (platelet count, prothrombin time, activated partial thromboplastin time (aPTT), fibrinogen, antithrombin, and prothrombin) as well as the decrease in ETP (before /after CBP) with both PRP and PDP at different TF concentrations**.** ETP values obtained by the different preparations were compared using a Kruskall-Wallis test both before and after surgery.

## Results

Surgery was uneventful as indicated by the following parameters: CPB duration (min): 64 (48-118); cross clamp time (min): 52 (25-90); 24 h blood loss (mL): 385 (170-750). Coagulation parameters measured before and after surgery are shown in Table 1.

**Table 1:** Patients’ coagulation parameters before and after surgery

	**Pre-operatively**	**Post-operatively**
**Platelet count (G/L)**	240 (109-337)	122 (67-201)
**prothrombin time (sec)**	12.9 (12.9-13.9)	16.5 (14.8-19.7)
**aPTT (sec)**	33 (29-59)	43 (34-58)
**Fibrinogen (g/L)**	4 (3.4-4.6)	2.3 (1.3-2.7)
**Prothrombin (%)**	85.5 (55-95)	57.5 (30-73)
**Antithrombin (%)**	97.5 (70-108)	64 (44-77)

Data are reported as median (min-max) of 10 patients. All decreases in platelet count, prothrombin time, aPTT, fibrinogen, prothrombin and antithrombin are statistically significant. *p* < 0.05 when comparing post-operating parameter to pre-operating parameter. *CPB: cardiopulmonary bypass, aPTT: activated partial thromboplastin time.*

Heparin neutralization with protamine was checked with thrombin time. No patient was transfused before blood sampling. Tranexamic acid was routinely used. No patient was transfused before the second blood sampling.

Thrombin generation was studied *in vitro* by CAT using citrated plasma in presence or absence of platelets.

As expected from the design of our study, no patient had abnormal bleeding, the maximal volume collected during the first 24 hours being 750 mL, and the lowest post-CPB ETP we observed, was greater than 1000 nM.min (with PRP).

Comparison of ETP from different experimental conditions by Kruskall-Wallis test did not show any significant difference before surgery (p = 0.0674). However after surgery ETP values were significantly different among preparations (p = 0.0009). Therefore we chose to compare ETP values before and after surgery for each preparation separately.

There was a statistically significant (Wilcoxon test) decrease in ETP (before /after CBP) with both PRP and PDP regardless the TF concentration. Our main finding was that CPB-induced coagulopathy was differently appreciated depending on the presence or absence of platelets. The percentage of variation between pre- and post-operative values of ETP was much lower with PRP (-3.9%) than with PDP (-39.6%) compared with a statistical Wilcoxon test (Table 2).

**Table 2:** Thrombin generation before and after CPB with PRP and PDP (at three TF concentrations)

	**ETP (nM.min)**		
	**Pre-operatively**	**Post-operatively**	**Change (%)**
**PRP (0.3 pM TF)**	1505 (1160-2464)	1491 (1087-2544)	-3.9 (4; -26.1)
**PDP (0.3 pM TF)**	1468 (1032-1913)	970 (500-1323)	-39.6 (13.9; -61.3)
**PDP (1 pM TF)**	1560 (1255-1928)	1083 (742-1990)	-24.4 (18.7; -52.7)
**PDP (5 pM TF)**	1723 (1338-2121)	1189 (810-2335)	-30,5 (28; -52.7)

Data are reported as median (min-max) of at least 8 patients (10 patients enrolled- a few missing data for technical reasons). All decreases in ETP are statistically significant. *p* < 0.05 when comparing ETP change with PRP to those with PDP in presence of 0.3 pM TF. *ETP: Endogenous Thrombin Potential. PDP: platelet-depleted plasma, PRP: platelet-rich plasma, TF: recombinant human tissue factor.*

The ETP pre-post variations observed with PRP were statistically significantly different compared to PDP in the presence of 0.3 pM TF, but not to PDP in the presence of 1 and 5 pM, probably because of a greater variations between the individual values. In our hands, 0.3 pM is the lowest TF concentration allowing reliable observation of thrombin generation and fully taking into account the ‘intrinsic’ tenase (anti-haemophilic factors).

## Discussion

Our results show that coagulopathy associated to CPB was observed, *in vitro*, not only with PPP but also with PRP. Our main new observation is that this coagulopathy was differently appreciated depending on the presence or absence of platelets.

The number of enrolled subjects (10 patients recruited in this study) was estimated to allow us to document the actual extent of hypocoagulability and to compare the different experimental settings.

As indicated above, no patient had abnormal bleeding. The lowest post-CPB ETP we observed, was greater than 1000 nM.min (with PRP); in one of our previous works [5], we reported the normal ETP to be 1579 ± 359 nM.min and for mild heamophiliacs the average value was 1060 ± 450 nM.min.

Our results concerning the decrease in ETP values with PDP after CPB were in agreement with previous studies [7,8] studied only with PPP and in presence of very higher TF concentration (20 pM) used in [7]. This suggests that the observed coagulopathy after CPB was not associated with the amplification phase of thrombin generation which depends on the TF concentration and is rather associated with the global phenomenon of thrombin generation.

Moreover, we did not find any consistent change in the duration of the initiation phase (by contrast to what has been reported in [8] “data not shown”). In addition, we have shown that this decrease in ETP values occurs also with PRP after CPB. Several mechanisms have been implicated in the haemostatic defects observed after CPB: consumption of coagulation factors and platelets, dilution, activation of fibrinolysis and transient platelet function defect [2,11-15].

Although we have not studied platelet function in the PRP of our patients, many other studies have described that platelet dysfunction is thought to result from many factors, such as contact with the synthetic surfaces of the extracorporeal circuit and hypothermia associated with bypass [2,11,12]. Many alterations have been reported, including α granule depletion and membrane glycoprotein decreases such as GPIIbIIIa [2,11,12]. No major alteration of procoagulant properties of platelets has been reported however, which is consistent with the slight decrease in ETP we observed with patients’ PRP. This explanation is supported by the observation of Reverter *et al.*[16] that a platelet dysfunction in patients of Glanzmann syndrome who lack GP IIb/IIIa on platelet surfaces results in a moderate impact on thrombin generation (21% less than normal platelets). They have also shown a moderate deficiency in thrombin generation (25%) in the presence of antibodies against GPIIbIIIa. Although this effect has not been described directly in the context of CPB, a significant decrease in the amount of membrane antigen for glycoproteins as IIb, and IIIa on circulating platelets following CPB has been reported [2]). In addition, the depletion of α granules, which contain GPIIbIIIa, has been reported in the context of CPB [2,12]. In addition, we suggest that other procoagulant actors would be present in PRP and not in PDP, capable of compensating plasma coagulopathy. This hypothesis is supported by the finding that procoagulant microparticles are generated during CPB, released not only from platelets but also from erythrocytes, monocytes, granulocytes and other cells, especially into pericardial blood [17]. Thus, these microparticles, eliminated with the centrifugation used to prepare PDP, might play, *in vitro*, a procoagulant role in coagulation with PRP, but not with PDP. Whatever the reason, it appears that a plasma hemostatic defect can be partly compensated by platelets [18]. However, the relation between these measurements, *in vitro*, and the clotting process, *in vivo,* is unknown.

In conclusion, our findings highlight that the experimental conditions are crucial in the study of *in vitro* thrombin generation in the context of CPB, the presence of platelets and microparticles being associated with a distinct pattern.

### Limitations of this study

Other experimental conditions for CAT had to be taken into account, for example, platelet count, the use of corn trypsin inhibitor (CTI) – to prevent artificial contact phase activation –, the concentration of TF; and the activated protein C system which would improve the relation to clinical outcome.

## Abbreviations

CPB: Cardiopulmonary bypass; CAT: Calibrated automated thrombography; ETP: Endogenous thrombin potential; PRP: Platelet-rich plasma; PPP: Platelet-poor plasma; PDP: Platelet-depleted plasma; PC: Phosphatidylcholine; PS: Phosphatidylserine; TF: Recombinant human tissue factor; aPTT: Activated partial thromboplastin time; CTI: Corn trypsin inhibitor.

## Competing interests

The authors declare that they have no competing interests.

## Authors' contributions

All authors read and approved the final manuscript.
